# C5 inhibition restores B cell homeostasis and humoral immunity in CHAPLE disease patients

**DOI:** 10.70962/jhi.20260042

**Published:** 2026-05-26

**Authors:** Azzeddine Tahiat, Reda Belbouab, Abdelghani Yagoubi, Sofija Buta Panov, Lorenzo Cuollo, Kamel Djenouhat, Dusan Bogunovic

**Affiliations:** 1 Department of Medical Biology, Rouiba Hospital, Algiers, Algeria; 2 Algiers University of Health Sciences, Algiers, Algeria; 3 Department of Pediatrics, Mustapha University Hospital, Algiers, Algeria; 4 Pediatric Gastroenterology, Centre Algérois de Pédiatrie, Algiers, Algeria; 5 Department of Pediatrics, Center for Genetic Errors of Immunity, Columbia University, New York, NY, USA

## Abstract

CHAPLE disease is a monogenic disorder in which CD55 deficiency drives gastrointestinal pathology. How CD55 deficiency affects adaptive immunity is unknown. Herein, molecularly, we characterize eight patients with genetically novel CD55 deficiency. Clinically, all patients developed early-onset protein-losing enteropathy, frequently complicated by thrombotic events, inflammatory bowel disease–like lesions, and recurrent respiratory infections. *Ex vivo*, immunophenotyping revealed disruption of the B cell compartment, marked by depletion of transitional B cells, expansion of CD21^lo^ B cells, and accumulation of class-switched memory B cells and plasmablasts, while T cell subsets were largely preserved. *In vivo*, eculizumab rapidly resolved intestinal pathology, normalized serum albumin and immunoglobulin levels, and re-established a normal B cell profile. Furthermore, C5 inhibition enabled effective humoral protection and optimal pneumococcal vaccine responses. Together, these findings establish complement inhibition as central to B cell homeostasis in CHAPLE disease.

## Teaser

CD55 deficiency drives complement hyperactivation, severe gastrointestinal pathology, and disrupted B cell homeostasis. Our findings show that complement inhibition in CHAPLE disease restores serum immunoglobulin levels, re-establishes a normal B cell profile, and enables effective humoral protection and optimal vaccine responses.

## Introduction

CD55, also known as decay-accelerating factor, is a key complement regulatory protein expressed on a broad range of cells, including endothelial cells lining blood and lymphatic vessels. By accelerating the decay of C3 and C5 convertases in the classical and alternative complement pathways, CD55 serves as a critical safeguard, protecting the endothelium from uncontrolled complement activation and preventing collateral tissue injury (1, 2). The critical role of CD55 was brought into sharp focus in 2017, with the identification of biallelic loss-of-function (LOF) mutations in the *CD55* gene as the molecular basis of an ultra-rare, autosomal recessive disorder characterized by complement hyperactivation, angiopathic thrombosis, respiratory infection susceptibility, and most prominently severe protein-losing enteropathy (PLE), now referred to as CHAPLE disease (OMIM #226300) (3). This discovery firmly established uncontrolled complement activation as the central pathogenic mechanism driving both the vascular and gastrointestinal manifestations of the disease, highlighting CD55 as a critical checkpoint in maintaining complement regulation and immune homeostasis (4).

CHAPLE disease is characterized by severe PLE, arising from primary intestinal lymphangiectasia (PIL) driven by complement-mediated attack to intestinal lymphatic vessels (4). Patients with CHAPLE disease present with chronic gastrointestinal symptoms, hypoalbuminemia-related edema, malabsorption with growth retardation, hypogammaglobulinemia, and increased susceptibility to infections (3, 4). CD55 deficiency is also associated with dysregulated coagulation and an elevated risk of thrombosis, similar to other complement-mediated disorders such as paroxysmal nocturnal hemoglobinuria and atypical hemolytic uremic syndrome (5). The central role of complement overactivation in disease pathogenesis has provided the rationale for complement inhibition therapies, which are now established in clinical practice (6, 7, 8). C5 inhibition with eculizumab demonstrated marked clinical and biological efficacy, including rapid resolution of gastrointestinal symptoms, normalization of serum protein levels, improved growth and quality of life, and sustained remission, accompanied by restoration of serum proteins, microbiota, endoscopic, and radiologic abnormalities (9).

Since its initial description, accumulating evidence has substantially advanced our understanding of CHAPLE disease (7, 8, 9, 10). Nevertheless, the systemic consequences of complement dysregulation on circulating lymphocyte homeostasis, subset composition, and functional competence, as well as the mechanisms driving infection susceptibility, remain poorly defined. Here, we report a cohort of eight patients with CHAPLE disease, comprehensively characterized at both clinical and molecular levels. We further delineate disease-associated alterations in B cell subsets and assess the impact of *in vivo* complement inhibition on B cell homeostasis and humoral immunity.

## Results

### Clinical phenotype

**Figure 1.**
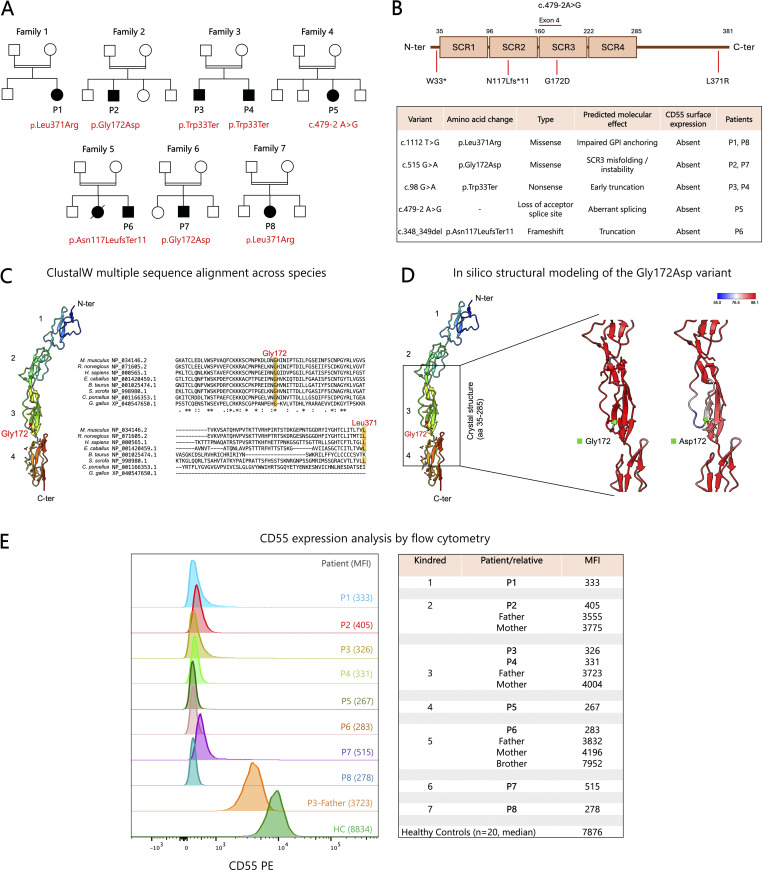
**Molecular characterization and cytometric analysis of CD55 deficiency in eight patients. (A)** Patients’ pedigrees, with the *CD55* variant shown for each patient. **(B)** Schematic representation of CD55 highlighting the location of variants, their predicted molecular effects, and their impact on surface CD55 expression. **(C)** ClustalW multiple sequence alignment across species, showing strict evolutionary conservation of Gly172 and Leu371, supporting the functional importance of these residues and the pathogenicity of the Gly172Asp and Leu371Arg substitutions. **(D)** Structural modeling of CD55 (aa 35–285) based on crystal structure (left, UniProt ID: 1OJV) and AlphaFold predictions (right, UniProt ID: P08174-4) reveals a localized alteration in conformational dynamics at residue 172. The wild-type SCR3 domain exhibits high-confidence features (red), whereas the p.Gly172Asp substitution affects the confidence in the predicted structure (blue). The legend shows the B-factor computed by ChimeraX. **(E)** FCM histograms showing complete loss of CD55 expression in all patients. Carrier parents (as exemplified by P3’s father in the overlaid histograms) display a monomodal, intermediate CD55 expression compared with controls. MFI values are shown for patients, carriers, and controls. aa, amino acid; HC, healthy control; SCR, short consensus repeat.

All eight patients, designated P1 through P8, originated from consanguineous families. Patients P3 and P4 were siblings, whereas P6 had a family history notable for a deceased sister who presented a comparable clinical phenotype (Table 1 and Fig. 1 A). Age at diagnosis ranged from 2 to 14 years (median: 6.5 years), with symptom onset occurring between 4 mo and 8 years (median: 24 mo). The clinical phenotype was uniformly dominated by early-onset PLE, primarily manifesting with gastrointestinal symptoms, including abdominal pain, chronic diarrhea, and vomiting. Chronic hypoalbuminemia was a consistent feature, accompanied by facial and/or extremity edema in all patients and ascites in five (62.5%). Thrombotic complications were observed in 6 patients (75%). P1 and P2 developed pulmonary embolism, with concomitant femoral vein thrombosis in P1 and cerebral venous thrombosis in P2. Budd–Chiari syndrome secondary to hepatic venous outflow obstruction occurred in P3, P4, and P7, whereas P5 presented with lower limb thrombosis. Five patients (62.5%), i.e., P1, P2, P5, P6, and P8, exhibited hypogammaglobulinemia, with three (P1, P5, and P8) presenting recurrent infections, predominantly affecting the respiratory tract. Six patients (75%) exhibited inflammatory bowel disease (IBD)–like lesions. Computed tomography (CT) and magnetic resonance (MR) enterography demonstrated segmental or multifocal bowel wall thickening with features of active inflammation, including parietal hyperemia and stenosis, predominantly involving the ileum and proximal jejunum. Complementary endoscopic and histologic assessments revealed lymphoid mucosal infiltrates in patients P1 and P8, and mucosal ulcers consistent with erosive Crohn’s disease–like colitis in patients P3 and P5. In patients P4 and P6, the affected intestinal segments were not amenable to endoscopic evaluation (Table 1).

**Table 1.** Demographic, clinical, and genetic characteristics of eight patients with CHAPLE disease

Characteristic	P1	P2	P3	P4	P5	P6	P7	P8
Ethnicity	Algerian	Algerian	Algerian	Algerian	Algerian	Syrian	Algerian	Algerian
***CD55* variant**								
Variant	c.1112 T>G	c.515 G>A	c.98 G>A	c.98 G>A	c.479-2 A>G	c.348_349del	c.515 G>A	c.1112 T>G
Amino acid change	p.Leu371Arg	p.Gly172Asp	p.Trp33Ter	p.Trp33Ter	–	p.Asn117LeufsTer11	p.Gly172Asp	p.Leu371Arg
Zygosity	Homozygous	Homozygous	Homozygous	Homozygous	Homozygous	Homozygous	Homozygous	Homozygous
**Demographic characteristics**								
Sex	Female	Male	Male	Male	Female	Male	Male	Female
Consanguinity in parents	Yes	Yes	Yes	Yes	Yes	Yes	Yes	Yes
Similar cases in sibling	No	No	Yes	Yes	No	Yes	No	No
Age at last follow-up (years)	7	8	7	4	16	5	9	12
Age at diagnosis (years)	5	7	6	2	14	2.5	8	11
Age at onset of symptoms (months)	13	4	24	20	60	24	48	96
**Manifestations of gastrointestinal disease**								
Chronic or recurrent diarrhea	Yes	Yes	Yes	Yes	Yes	Yes	Yes	Yes
Abdominal pain	Yes	Yes	Yes	Yes	Yes	Yes	Yes	Yes
Vomiting	Yes	Yes	Yes	Yes	No	Yes	Yes	Yes
**Features of PLE**								
Hypoalbuminemia	Yes	Yes	Yes	Yes	Yes	Yes	Yes	Yes
Facial or extremity edema	Yes	Yes	Yes	Yes	Yes	Yes	Yes	Yes
Ascites	No	No	Yes	Yes	Yes	No	Yes	Yes
**Malabsorption features**								
Growth retardation	Yes	No	Yes	Yes	Yes	Yes	Yes	Yes
Anemia	Yes	Yes	Yes	Yes	Yes	No	Yes	Yes
Micronutrient deficiency	Yes	Yes	Yes	Yes	Yes	Yes	Yes	Yes
**Features of thrombotic disease**								
Thrombosis	Femoral vein thrombosis, pulmonary embolism	Cerebral venous thrombosis, pulmonary embolism	Budd–Chiari syndrome	Budd–Chiari syndrome	Lower limb thrombosis	No	Budd–Chiari syndrome	No
**Imaging findings**								
Bowel wall thickening	Yes	No	Yes	Yes	Yes	Yes	No	Yes
Inflammatory changes	Yes	No	Yes	Yes	Yes	Yes	No	Yes
**Endoscopic findings**								
Mucosal ulcers	No	No	Yes	No	Yes	No	No	No
Mucosal nodules	No	No	No	No	Yes	No	No	Yes
Lymphoid infiltrates in mucosa	Yes	No	Yes	No	Yes	No	No	Yes
**Infectious disease**								
Hypogammaglobulinemia	Yes	Yes	No	No	Yes	Yes	No	Yes
Recurrent respiratory infections	Yes	No	No	No	Yes	No	No	Yes
Additional features	Hepatomegaly	Hypothyroidism, asthma	Hepatomegaly, cirrhosis, asthma	Hepatomegaly, cirrhosis	​	​	Hepatomegaly, cirrhosis	Hepatomegaly, splenomegaly, mesenteric lymphadenopathy, asthma
Treatment	Supportive and symptomatic management	Supportive and symptomatic management	Supportive and symptomatic management	Supportive and symptomatic management	Eculizumab	Eculizumab	Supportive and symptomatic management, liver transplantation	Eculizumab
Alive/Deceased	Deceased at 7 years old (y/o)	Deceased at 8 y/o	Deceased at 7 y/o	Deceased at 4 y/o	Alive	Alive	Alive	Alive

### LOF mutations in *CD55* and surface expression analysis

Whole-exome sequencing (WES) identified five novel variants in the *CD55* gene (Table 1; and Fig. 1, B and C). Patients P1 and P8 harbored the same homozygous missense variant (c.1112T>G; p.Leu371Arg), affecting a highly conserved hydrophobic leucine in the C-terminal region of CD55, immediately upstream of the glycosylphosphatidylinositol (GPI) anchor signal. Patients P2 and P7 carried another homozygous missense variant (c.515G>A; p.Gly172Asp) within the third extracellular short consensus repeat (SCR3) domain. Replacement of the small, flexible glycine with a negatively charged aspartic acid is predicted to affect the domain structure and increase local rigidity, as predicted by DynaMut (ΔΔG: 1.209 kcal/mol) (11) (Fig. 1 D). A homozygous nonsense variant (c.98G>A; p.Trp33Ter) was identified in siblings P3 and P4, resulting in a premature termination codon at position 33. In patient P5, a homozygous splice-site mutation (c.479-2A>G) occurring two nucleotides upstream of the canonical splice acceptor site of exon 4 was identified. Finally, the c.348_349del variant (p.Asn117LeufsTer11) identified in patient P6 induces a frameshift within exon 3, producing a premature termination codon 11 residues downstream.

All patients exhibited a complete loss of CD55 expression, with median fluorescence intensity (MFI) values comparable to those of the isotype control. Reference MFI values were established from a cohort of 20 healthy controls. The assessment of CD55 expression in the carrier parents of three patients revealed an intermediate pattern, with expression levels (MFI) representing ∼50% of those observed in healthy controls supportive of lack of haploinsufficiency clinically observed (Fig. 1 E).

### Serum protein levels and lymphocyte phenotype

#### Serum albumin, complement, and immunoglobulin levels

**Figure 2.**
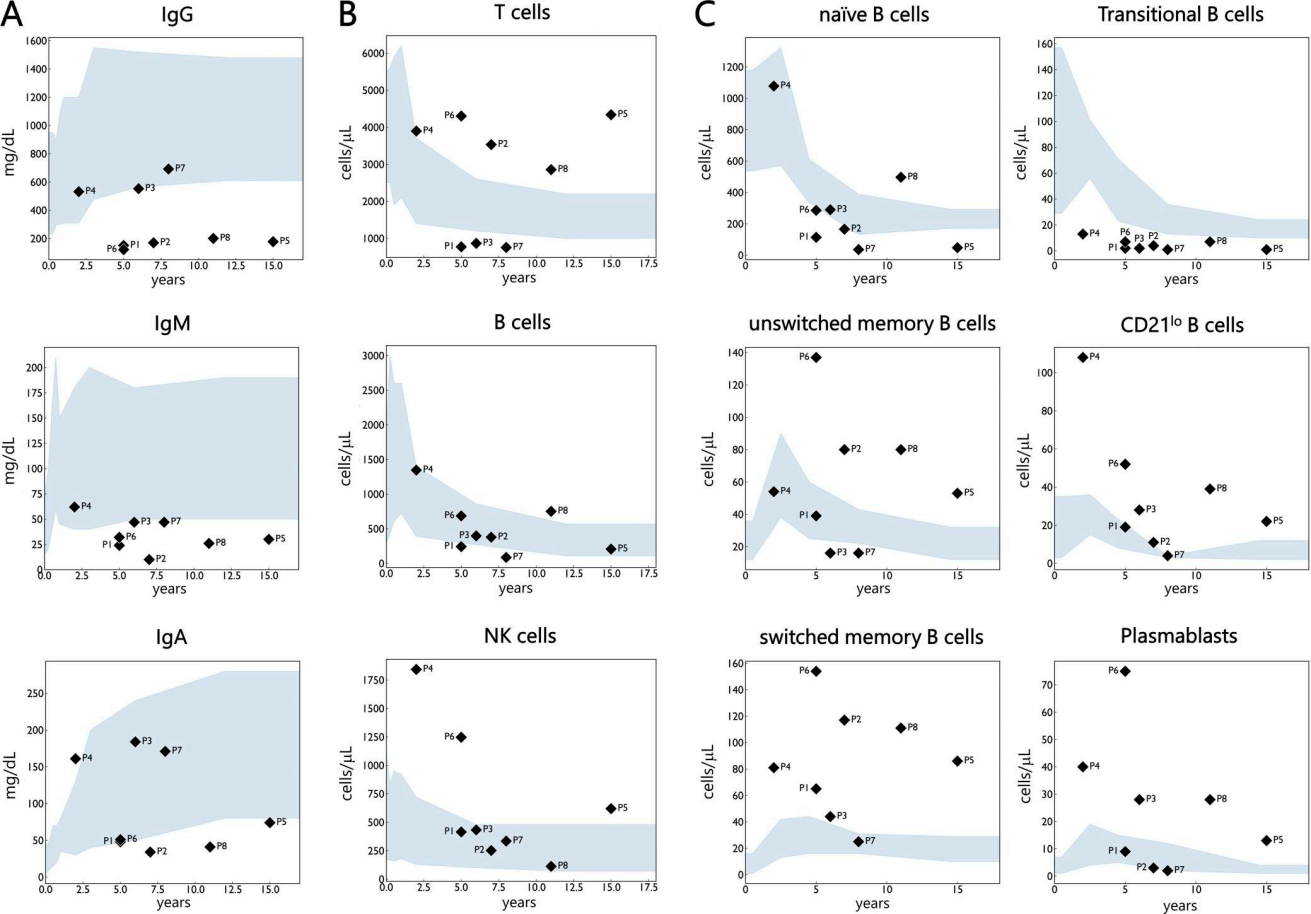
**Serum immunoglobulin levels and lymphocyte subset counts in eight patients with CHAPLE disease. (A)** Serum immunoglobulin levels. **(B)** T, B, and NK cell counts. **(C)** B cell subset counts. Grey-blue shaded areas indicate age-matched normal ranges, with values outside considered abnormal (12, 13).

Chronic hypoalbuminemia was observed in all patients, with serum albumin concentrations at diagnosis ranging from 12 to 27 g/L (median: 20 g/L). Low C3 level was observed in two patients (P5 and P8), whereas decreased C4 was noted in P2. Decreased IgG levels were observed in five patients (62.5%), ranging from 122 to 692 mg/dl, with a median of 189.5 mg/dl. IgM levels were reduced in six patients (75%), with a median of 31 mg/dl (range: 10–62). IgA levels were moderately to slightly decreased in three patients (37.5%) and elevated in one (P4) (median: 62.5 mg/dl; range: 34–184) (Fig. 2 A and Table S1).

#### Lymphocytes phenotype

**Figure S1.**
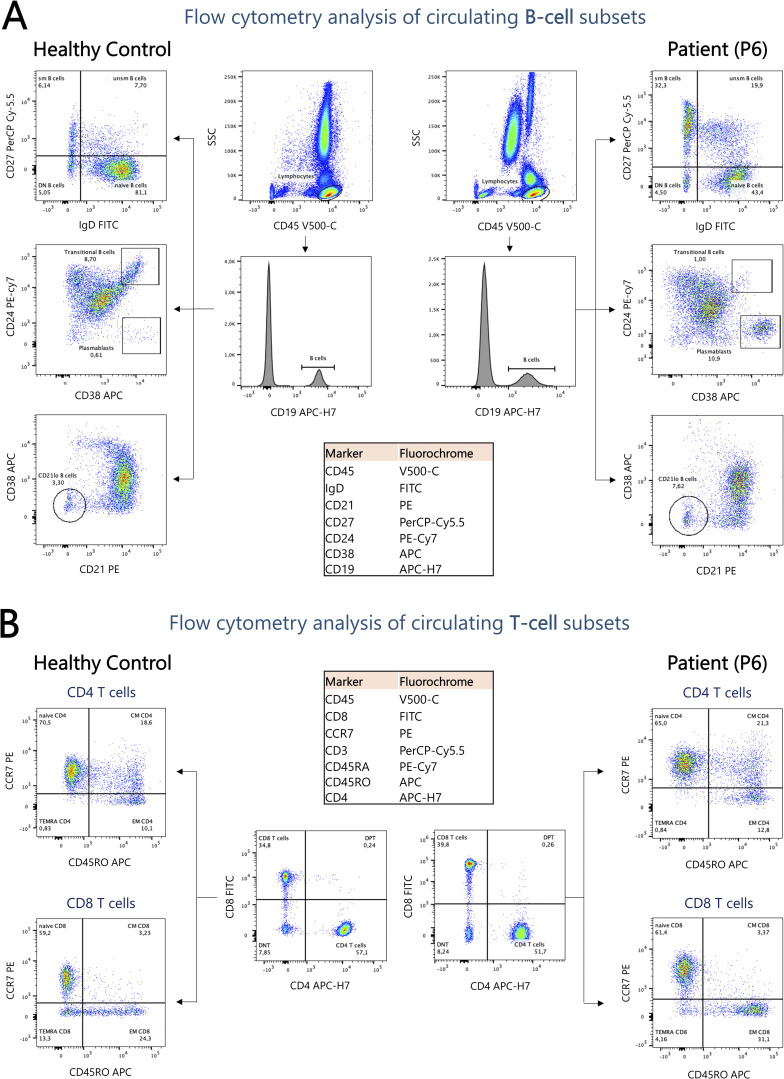
**Markers and gating strategy for B and T cell immunophenotyping. (A)** Representative FCM dot plots illustrating the analysis of B cell subsets in patient P6 (5 years) compared with an age-matched healthy control. P6 displays increased frequencies of switched and unswitched memory B cells, as well as plasmablasts, alongside a marked reduction in transitional B cells. **(B)** Analysis of T cell subsets in P6 did not reveal significant differences compared with the healthy control. CM, central memory; DN, double-negative; DPT, double-positive T cells; EM, effector memory; sm, switched memory; TEMRA, terminally differentiated effector memory T cells re-expressing CD45RA; unsm, unswitched memory.

Detailed immunophenotypic analysis of T and B cell compartments was performed in all patients (Table S1 and Fig. S1). Total T cell counts were normal to mildly elevated in five patients and mildly reduced in three. Subset analysis revealed reduced naïve CD4^+^ and CD8^+^ T cells in three patients (37.5%), whereas memory CD4^+^ and CD8^+^ T cell subsets were expanded in four (50%). Elevated natural killer (NK) cell counts were noted in three patients (37.5%) (Fig. 2 B). Strikingly, all patients exhibited profound perturbations in circulating B cell compartment. Naïve CD27^^−^^IgD^+^ B cells were reduced in four patients (50%), while class-switched memory B cells were markedly expanded in all patients, with absolute counts above the normal reference range in seven (87.5%). Unswitched memory B cells were also increased in four patients (50%). Transitional B cells were profoundly diminished in all patients, by up to 15-fold in some cases, accounting for only 0.4–1% of circulating B cells compared with a reference range of 4–16% (12). CD21^lo^ B cells, also referred to as age-associated B cells (ABCs) and previously linked to autoimmune and autoinflammatory conditions, immunodeficiency, and human immunodeficiency virus infection (14, 15, 16, 17), were increased in all patients (either in frequency or absolute numbers). Finally, circulating plasmablasts counts were increased in five patients (62.5%), namely P3, P4, P5, P6, and P8 (Fig. 2 C).

### Treatment and outcome

All patients received supportive and symptomatic care, including intravenous albumin infusions and anticoagulation for thrombotic events. Patients P5, P6, and P8 were treated with eculizumab (Elizaria), whereas P7 underwent liver transplantation. Regarding clinical outcomes, P1, P2, P3, and P4 have died between 4 and 8 years, while P5, P6, P7, and P8 remain alive (Table 1).

### Clinical and biological response to eculizumab therapy

Off-label eculizumab therapy was initiated in three patients, with post-treatment follow-up durations of 45 wk (P5), 8 wk (P6), and 29 wk (P8). All patients exhibited a rapid and robust clinical response, marked by prompt resolution of peripheral edema and gastrointestinal manifestations, including abdominal pain, diarrhea, and vomiting, and a concomitant improvement in overall quality of life. These clinical improvements were paralleled by normalization of serum protein parameters. Hypoalbuminemia resolved in all treated patients, with serum albumin levels increasing from 19 to 45 g/L in P5, from 12 to 41 g/L in P6, and from 23 to 45 g/L in P8. In parallel, hypogammaglobulinemia was corrected in all three patients, accompanied by a marked reduction in infectious burden, most notably respiratory infections in patients P5 and P8 (Table 2).

**Table 2.** Serum protein levels and lymphocyte phenotype before and after eculizumab therapy

Parameter	P5		P6		P8	
	Pre-eculizumab	45 wk after eculizumab	Pre-eculizumab	8 wk after eculizumab	Pre-eculizumab	29 wk after eculizumab
Age at evaluation, years	15	16	5	5	11	12
Albumin, g/L	19	45	12	41	23	45
**Complement proteins, mg/dl**						
C3	82	99	89	130	82	146
C4	20	24	22	46	21	37
**Immunoglobulins, mg/dl**						
IgG	178	992	122	981	201	1,210
IgM	30	228	32	122	26	203
IgA	74	80	51	183	41	223
**Lymphocyte phenotyping**						
Lymphocytes, cells/μl	5,166	2,855	6,237	6,290	3,758	2,781
**T-B-NK enumeration, cells/μl**						
CD3^+^ T cells	4,340	2,113	4,304	4,529	2,856	2,113
CD4^+^ T cells	2,170	1,256	2,245	2,013	1,654	1,279
CD8^+^ T cells	2,015	742	1,559	1,950	940	612
CD4/CD8 ratio	1.08	1.69	1.44	1.03	1.76	2.09
CD19^+^ B cells	207	343	686	692	752	473
NK cells	620	400	1,247	1,069	113	195
**Extended CD4** ^**+**^ **T cell phenotyping, % of CD4 (cells/μl)**						
CD4^+^CD45RA^+^	48 (1,042)	35 (440)	66 (1,482)	62 (1,248)	51 (844)	47 (601)
Naïve CD4^+^ T cells (CD45RA^+^CCR7^+^)	46 (998)	35 (440)	65 (1,459)	62 (1,248)	50 (827)	46 (588)
Memory CD4^+^ T cells (CD45RO^+^)	52 (1,128)	65 (816)	34 (763)	38 (765)	49 (810)	53 (678)
**Extended CD8** ^**+**^ **T cell phenotyping, % of CD8 (cells/μl)**						
CD8^+^CD45RA^+^	58 (1,169)	55 (408)	66 (1,029)	60 (1,170)	49 (461)	52 (318)
Naïve CD8^+^ T cells (CD45RA^+^CCR7^+^)	37 (746)	32 (237)	61 (951)	54 (1,053)	32 (301)	32 (196)
Memory CD8^+^ T cells (CD8^+^CD45RO^+^)	42 (846)	45 (334)	34 (530)	40 (780)	51 (479)	48 (294)
**Extended CD19** ^**+**^ **B cell phenotyping, % of CD19 (cells/μl)**						
Naïve B cells (CD27^–^IgD^+^)	23.2 (48)	80.7 (277)	41.7 (286)	61.4 (425)	66.1 (497)	68.8 (325)
Unswitched memory B cells (CD27^+^IgD^+^)	25.5 (53)	10.4 (36)	19.9 (137)	18.7 (129)	10.6 (80)	11.4 (54)
Switched memory B cells (CD27^+^IgD^–^)	41.5 (86)	6.0 (22)	22.5 (154)	11.4 (79)	14.7 (111)	12.9 (61)
Transitional B cells (CD24^++^CD38^++^)	0.4 (1)	7.6 (26)	1.0 (7)	5.9 (41)	0.9 (7)	5.8 (27)
CD21^lo^ B cells (CD21^low^CD38^low^)	10.7 (22)	2.1 (7)	7.6 (52)	5.3 (37)	5.2 (39)	3.1 (15)
Plasmablasts (CD24^–^CD38^++^)	6.2 (13)	1.8 (6)	10.9 (75)	5.9 (41)	3.7 (28)	3.2 (15)

### Reversible circulating B cell abnormalities after eculizumab therapy

**Figure 3.**
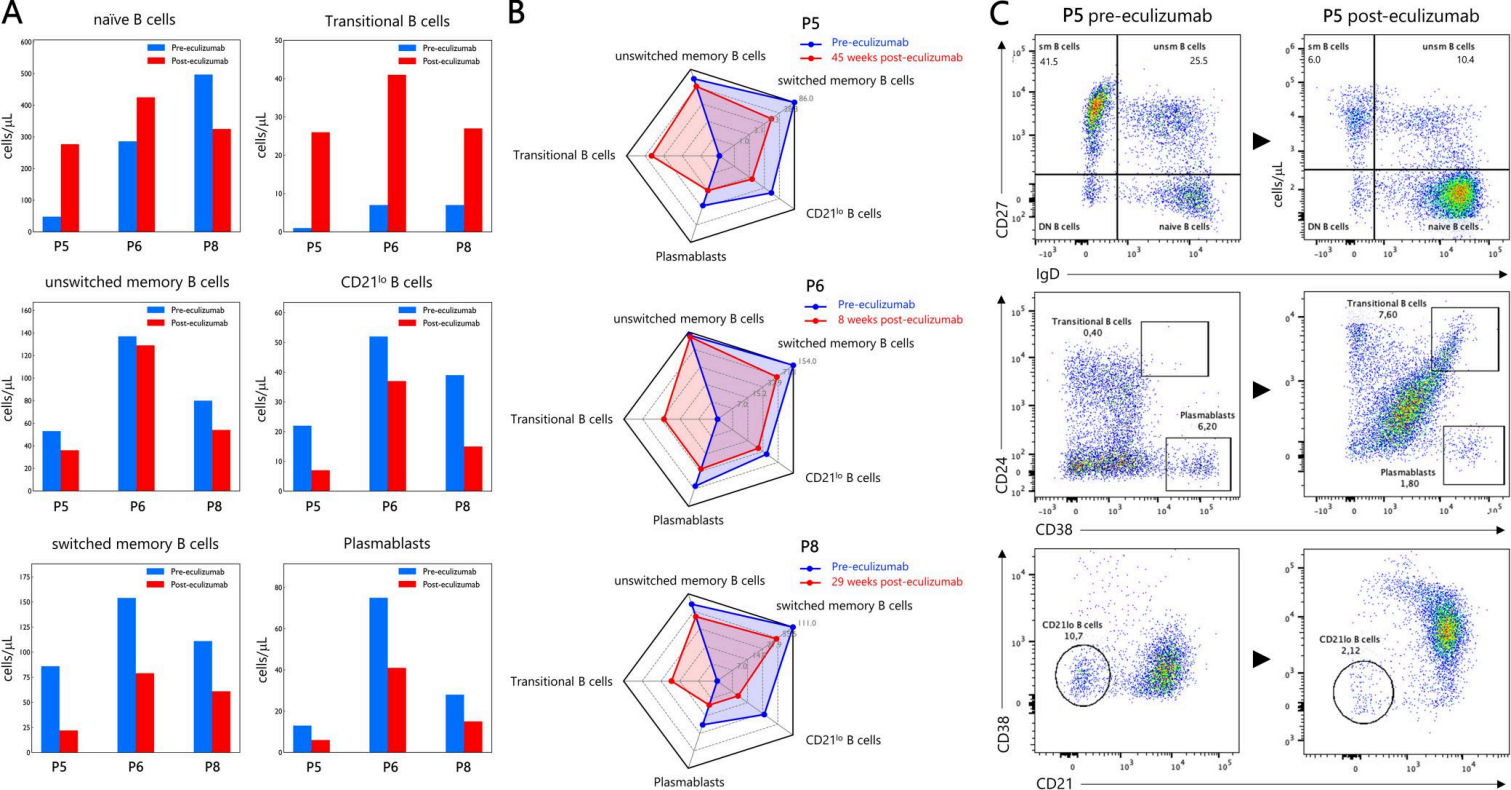
**B cell subset frequencies and counts before and after eculizumab therapy in patients P5, P6, and P8. (A and B)** B cell subset distributions before and after eculizumab treatment are shown as bar graphs (A) and radar charts (B). Eculizumab treatment was associated with an increase in transitional B cell frequencies and absolute counts and a marked reduction in CD21^lo^ B cells, switched memory B cells, and plasmablasts in all three patients. **(C)** These changes are further illustrated by FCM dot plots depicting the distribution of B cell subsets before and after eculizumab therapy in patient P5. sm, switched memory; unsm B cells, unswitched memory.

Comparative analysis of lymphocyte subsets before and after eculizumab therapy revealed no significant changes in T cell subsets. In contrast, the systemic B cell compartment underwent marked post-treatment remodeling, most prominently characterized by the restoration of circulating transitional B cells. Transitional B cells increased substantially in both frequency and absolute number, reaching normal ranges in all treated patients. After eculizumab therapy, they increased from 0.4% (1 cell/μl) to 7.6% (26 cells/μl) in P5, from 1.0% (7 cells/μl) to 5.9% (41 cells/μl) in P6, and from 0.9% (7 cells/μl) to 5.8% (27 cells/μl) in P8 (Table 2). In parallel, a consistent reduction in both the frequency and absolute counts of CD21^lo^ B cells was observed across all three patients. Concordantly, class-switched memory B cells and plasmablasts also declined following treatment in all patients (Fig. 3, A–C).

### Dynamics of pneumococcal serotype–specific IgG concentrations following vaccination and eculizumab therapy

**Figure 4.**
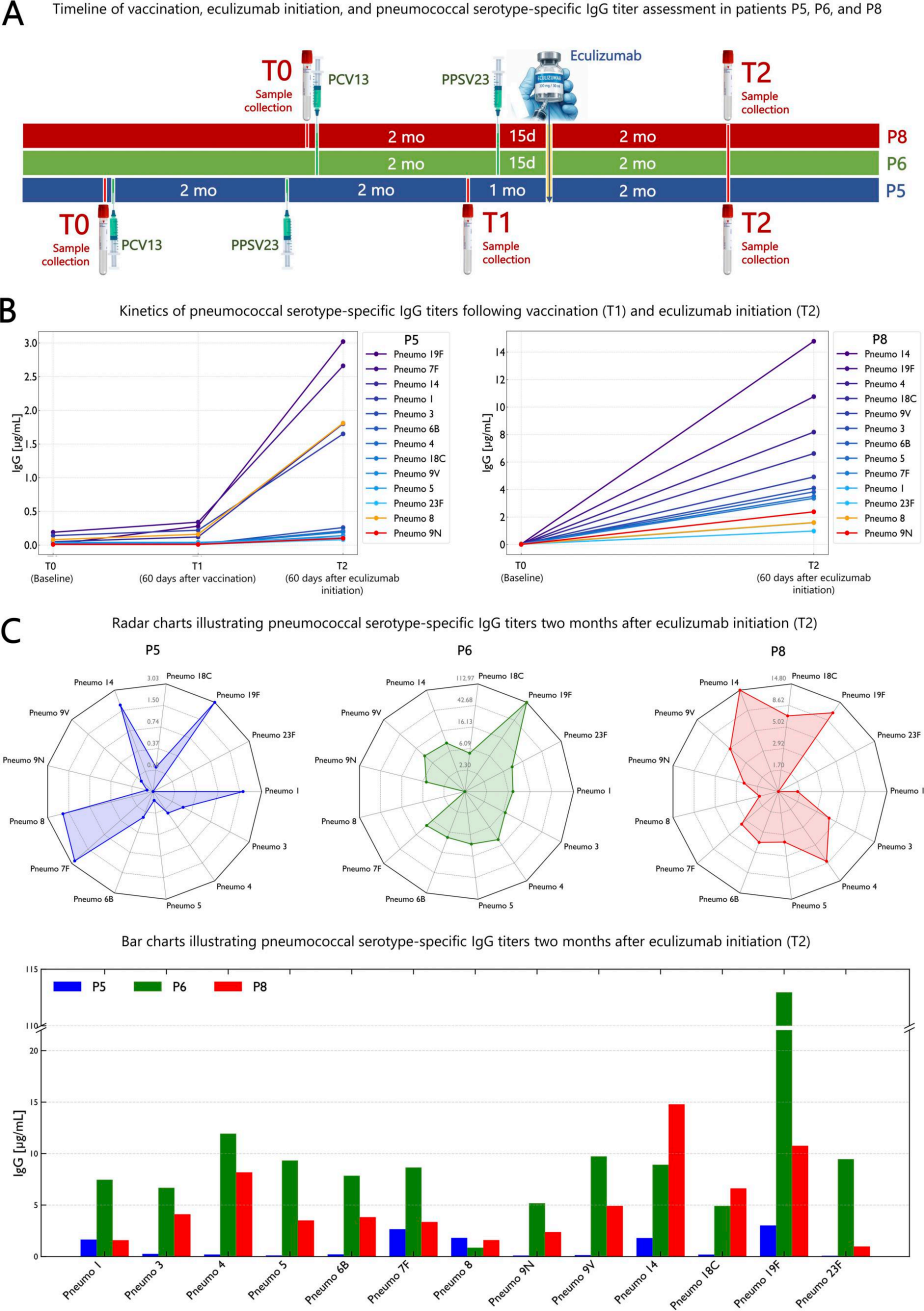
**Impact of eculizumab timing on serotype-specific IgG responses to PCV13 and PPSV23 vaccination. (A)** Visual timeline of vaccination, eculizumab initiation, and pneumococcal serotype–specific IgG titer assessment in patients P5, P6, and P8. **(B)** Kinetics of pneumococcal serotype–specific IgG concentrations following sequential PCV13 and PPSV23 vaccination and eculizumab therapy. The left panel shows longitudinal three-point curves of IgG concentrations at T0 (baseline), T1 (60 days after sequential PCV13 and PPSV23 vaccination, prior to eculizumab), and T2 (60 days after eculizumab initiation) in patient P5. The right panel shows two-point curves of IgG titers at baseline (T0) and 60 days after eculizumab initiation (T2) in patient P8. T cell–dependent PCV13 serotypes (1, 3, 4, 5, 6B, 7F, 9V, 14, 18C, 19F, and 23F) are shown in shades of blue, whereas T cell–independent PPSV23 serotypes 8 and 9N are shown in orange and red, respectively. **(C)** IgG concentrations against individual pneumococcal serotypes in patients P5, P6, and P8 2 mo after eculizumab treatment, illustrated as radar charts and bar charts. PCV13: 13-valent pneumococcal conjugate vaccine; PPSV23: 23-valent pneumococcal polysaccharide vaccine.

Following sequential 13-valent pneumococcal conjugate vaccine (PCV13) and 23-valent pneumococcal polysaccharide vaccine (PPSV23) vaccination, pneumococcal serotype-specific IgG titers varied according to serotype and the timing of eculizumab administration (Table S2). In patient P5, IgG concentrations measured 60 days after vaccination showed modest increases for both T cell–dependent PCV13 serotypes and T cell–independent PPSV23 serotypes (8 and 9N), remaining below protective thresholds (<1.3 µg/ml). Two months after initiation of eculizumab therapy (T2), IgG concentrations for T cell–dependent serotypes partially recovered, reaching protective levels for 4 of 11 serotypes: 1 (1.65 µg/ml), 7F (2.66 µg/ml), 14 (1.80 µg/ml), and 19F (3.02 µg/ml). Regarding T cell–independent serotypes, a modest increase for serotype 8 (1.81 µg/ml) and nearly undetectable titers for 9N (0.10 µg/ml) (Fig. 4, A and B). In patients 6 and 8, who received eculizumab shortly after sequential PCV13 and PPSV23 vaccination, serotype-specific IgG measurements performed 2 mo post-therapy initiation revealed adequate titers. Patient P6 exhibited protective IgG concentrations across all T cell–dependent serotypes (1, 3, 4, 5, 6B, 7F, 9V, 14, 18C, 19F, 23F; range: 4.92–112.96 µg/ml) and partial responses for T cell–independent serotypes 8 (0.86 µg/ml) and 9N (5.18 µg/ml). Similarly, patient P8 displayed protective IgG concentrations for most T cell–dependent serotypes (1, 3, 4, 5, 6B, 7F, 9V, 14, 18C, 19F; range: 1.59–14.79 µg/ml) and modest responses for T cell–independent serotypes 8 (1.60 µg/ml) and 9N (2.38 µg/ml) (Fig. 4 C). Taken together, these findings indicate that C5 inhibition in CD55 deficiency not only resolves PLE but also restores humoral immunity and vaccine efficacy, thereby enhancing infectious disease readiness.

## Discussion

CHAPLE disease is a unique monogenic disorder in which uncontrolled complement activation, due to CD55 deficiency, primarily drives intestinal pathology, manifesting as PLE and its associated complications (10). The clinical phenotype of our cohort closely mirrors that originally described by Ozen et al. with PLE as the defining hallmark (3). Gastrointestinal manifestations were universal, consistently accompanied by hypoalbuminemia and edema. Thrombotic complications affected 75% of patients, encompassing pulmonary embolism, cerebral venous thrombosis, Budd–Chiari syndrome, and lower limb thrombosis. Hypogammaglobulinemia was observed in five patients (62.5%), with recurrent pneumonia documented in three (37.5%). Comparable respiratory infections were reported in 5 of 11 patients (45.5%) in the Turkish cohort, highlighting the functional consequences of impaired humoral immunity (3). Another notable finding was the predominance of IBD-like inflammatory lesions. Six patients (75%) demonstrated segmental or multifocal bowel wall thickening and inflammatory changes on imaging, while endoscopic and histologic evaluation revealed lymphoid mucosal infiltrates in four patients, with mucosal ulcers observed in two. Comparable inflammatory lesions were reported by Ozen et al. who documented endoscopic abnormalities and/or mucosal lymphoid infiltrates in 6 of 11 patients (3). The radiologic resolution of inflammatory lesions and endoscopic healing of ulcers following C5 inhibition with eculizumab, as reported by the same team, firmly implicate uncontrolled complement activation as a central pathogenic driver of the IBD-like phenotype in CHAPLE disease (9). Collectively, these observations highlight the essential role of CD55 in maintaining intestinal immune homeostasis, extending beyond its canonical function in protecting lymphatic endothelium from complement-mediated injury, and position CD55 deficiency as a unique monogenic model linking dysregulated complement activation to bowel inflammation (18).

Five novel homozygous *CD55* variants were identified in our cohort, with p.Leu371Arg and p.Gly172Asp recurring in unrelated patients, suggesting a potential founder effect (Fig. 1, A–C). Despite their mechanistic diversity, including missense substitutions (c.1112T>G, p.Leu371Arg; c.515G>A, p.Gly172Asp), nonsense (c.98G>A, p.Trp33Ter), frameshift (c.348_349del, p.Asn117LeufsTer11), and splice-site (c.479-2A>G) mutations, all variants resulted in complete loss of CD55 surface expression. The two missense variants exhibit distinct molecular consequences. p.Leu371Arg affects a highly conserved hydrophobic residue immediately upstream of the GPI-anchor signal, likely disrupting recognition and attachment of the GPI-anchor and thereby preventing membrane localization. p.Gly172Asp, located within the SCR3 domain, replaces a small, flexible glycine with a larger, negatively charged aspartic acid, possibly perturbing local folding and domain stability, impairing trafficking, and promoting premature degradation. In contrast, nonsense, frameshift, and splice-site variants introduce premature termination codons or aberrant transcripts, triggering nonsense-mediated decay and eliminating all functional domains.

Our study underscores that CHAPLE disease is a potentially life-threatening disorder with pronounced clinical heterogeneity. Four patients died between 4 and 8 years of age, reflecting early-onset PLE, severe malnutrition, and rapidly progressive disease, whereas others presented later with a milder, more indolent course. This interindividual variability does not appear to correlate with the nature of the underlying variants, as the same missense mutation (p.Leu371Arg) resulted in markedly divergent disease severity between patients P1 and P8 (Table 1). Additional factors likely influence disease course and severity, including environmental exposures, microbiome composition, diet, infections, lifestyle, concomitant conditions, and potentially modifier genes (4). The introduction of complement inhibition therapy has markedly transformed the natural history of CHAPLE disease (7, 8). In particular, eculizumab, a humanized monoclonal antibody targeting C5 to block formation of the terminal complement complex (C5b-9) and prevent complement-mediated tissue injury, has proven highly effective (9). Consistent with previous reports, therapy with eculizumab in three patients led to rapid and striking clinical improvement, with enhanced quality of life and normalization of serum proteins, including albumin and immunoglobulins, reflecting effective correction of PLE and associated humoral deficits (6, 9).

A primary aim of this study was to delineate the impact of CD55-associated intestinal pathology on the circulating T and B cell compartments. By targeting the central pathogenic driver of CHAPLE disease, namely uncontrolled complement activation, eculizumab provided a unique opportunity to assess the reversibility of immunophenotypic abnormalities, thereby establishing a direct causal link between CD55 deficiency and the observed lymphocyte perturbations. PIL, the signature feature of CHAPLE disease, could plausibly drive the selective depletion of specific lymphocyte subsets. Other forms of PIL are consistently associated with preferential loss of naïve CD4^+^ T cells (19, 20, 21, 22, 23), with analogous, albeit less-characterized, alterations in B cell populations (19). In our cohort, however, depletion of naïve CD4^+^ T cells was mostly not observed, whereas transitional B cells were strikingly and uniformly lost. These findings indicate that transitional B cells are particularly sensitive to complement-driven intestinal pathology, although gut integrity alone cannot fully explain their selective loss.

Recent advances in our understanding of human B cell ontogeny indicate that B cells continuously exit the bone marrow as transitional type 1 (T1) cells, from which all peripheral B cell subsets are derived. Upon maturation to the T2 stage, transitional B cells undergo a critical developmental bifurcation. IgM^hi^ T2 cells selectively express the α4β7 integrin and are preferentially recruited to gut-associated lymphoid tissue (GALT) and spleen via interactions with MAdCAM-1, whereas IgM^lo^ T2 cells express CCR7 and L-selectin, directing their homing to systemic lymphoid tissues such as lymph nodes and tonsils (24, 25). These observations highlight a central role for intestinal secondary lymphoid structures in the peripheral maturation of transitional B cells, particularly along the IgM^hi^ T2 pathway. In CHAPLE disease, chronic complement activation drives lymphangiectasia, persistent intestinal inflammation, and architectural distortion of GALT (10), creating an environment that impairs IgM^hi^ T2 maturation. This dysfunction likely disrupts local B cell differentiation within the gut, compromising the establishment of a balanced systemic B cell compartment.

Strikingly, the near-complete loss of transitional B cells was paradoxically accompanied by a pronounced expansion of CD21^lo^ B cells and an accumulation of late-stage populations, including class-switched memory B cells and plasmablasts. This constellation argues strongly for an active skewing of B cell fate rather than simple numerical attrition. We posit that chronic complement hyperactivation acts as a central upstream mediator of this imbalance (26, 27, 28). Elevated C3 and C5 fragments, together with additional inflammatory cues, may aberrantly activate transitional IgM^hi^ T2 B cells, biasing their differentiation toward inflammatory subsets such as CD21^lo^ ABCs, a population significantly expanded in all our patients and strongly associated with chronic inflammation and autoimmunity (14, 15, 16, 17, 29, 30). In parallel, IgM^lo^ T2 transitional B cells, which under physiological conditions are destined for maturation within systemic lymphoid organs (25), may be diverted under inflammatory conditions toward extrafollicular differentiation pathways, further contributing to the overrepresentation of ABCs, class-switched memory B cells, and plasmablasts (31). Together, these processes define a feed-forward pathological loop in CHAPLE disease, whereby uncontrolled complement activation drives intestinal inflammation and lymphatic dysfunction, which in turn distort B cell maturation and homeostasis, favoring terminal differentiation at the expense of immature transitional compartments. The centrality of complement in this cascade is underscored by our *ex vivo* and longitudinal observations following eculizumab therapy. Complement inhibition was associated with rapid restoration of circulating transitional B cells within less than 8 wk in patient P6, alongside contraction of CD21^lo^ B cells, class-switched memory B cells, and plasmablasts (Fig. 3, A–C). This rapid reversibility provides compelling functional evidence that B cell dysregulation in CHAPLE disease is not fixed, but dynamically linked to complement-mediated intestinal pathology.

Post-therapy follow-up revealed that complement blockade in patients P6 and P8 was associated with a marked reduction in the frequency of respiratory infections. To further delineate the impact of eculizumab on humoral immunity and infection susceptibility, we assessed pneumococcal serotype–specific IgG responses to vaccination before and after therapy. These analyses highlighted the importance of the timing of complement inhibition for effective T cell–dependent, PCV13-elicited humoral immunity. Patients P6 and P8, in whom eculizumab was initiated shortly after vaccination, maintained optimal post-therapy IgG titers across most pneumococcal serotypes. In contrast, delayed initiation of complement blockade in patient P5 was associated with weak to null IgG concentrations, with only partial recovery following eculizumab treatment, likely reflecting residual antibody production by long-lived plasma cells. Collectively, these findings indicate that B cell priming, T follicular helper cell–dependent cooperation, and germinal center–driven immunological memory formation are substantially preserved in CHAPLE disease. However, effective humoral protection appears to depend critically on timely control of complement-mediated antibody loss. By contrast, IgG responses to T cell–independent pneumococcal serotypes (8 and 9N) were consistently weak to moderate, reflecting the predominantly IgM-driven nature of polysaccharide-specific immune responses and their limited class switching to IgG. Moreover, the severe depletion of circulating transitional B cells, compounded by chronic intestinal inflammation, is likely to further compromise polysaccharide-specific immunity. Emerging evidence supports a critical role for the GALT–splenic axis in the maturation of transitional B cells into marginal zone (MZ) B cells and in the generation of T cell–independent humoral responses (25, 32). By the same token, studies in CD55-deficient mice demonstrate that CD55 loss disrupts MZ B cell homeostasis, increases apoptosis, and impairs IgG responses *in vivo*, underscoring its essential role in MZ B cell survival and function (33).

Taken together, these findings indicate that a prolonged interval between vaccination and complement blockade may be counterproductive in CHAPLE disease, as ongoing complement-mediated losses of proteins and immunoglobulins can diminish vaccine-induced antibody titers and compromise protective immunity. Accordingly, vaccines against *Neisseria meningitidis*, *Streptococcus pneumoniae*, and *Haemophilus influenzae* type B, administered as part of standard pre-eculizumab prophylaxis, should be given in close temporal proximity to eculizumab initiation. Polysaccharide vaccines, in particular, elicit suboptimal IgG responses prior to complement blockade, further supporting post-eculizumab vaccination to enhance vaccine-driven humoral protection.

Although these conclusions should be nuanced in light of the limited sample size of our study, our findings shed light on the impact of intestinal immunoglobulin loss on vaccine effectiveness, underscoring the importance of accounting for this mechanism in pre-therapeutic vaccination strategies in CHAPLE disease. Moreover, our data provide insight into a potential intrinsic defect in anti-polysaccharide immunity, possibly linked to perturbations of the gut–spleen axis and to CD55 function as a membrane receptor, independent of protein loss, thereby suggesting an additional layer of complexity underlying infection susceptibility in this condition. Collectively, these observations highlight the need for further investigation in larger, multicenter cohorts, ideally complemented by studies of local immune responses at the intestinal mucosal level.

In summary, this study shows that, beyond local gut injury, uncontrolled complement activation due to CD55 deficiency disrupts B cell homeostasis, depleting transitional B cells and expanding late-stage subsets, including class-switched memory B cells and plasmablasts. Complement blockade with eculizumab rapidly restores transitional B cell frequencies and reduces the inflammatory CD21^lo^ subset, reestablishing a more homeostatic B cell compartment. This phenotypic improvement is accompanied by normalization of serum immunoglobulins, restoring effective and protective humoral immunity and optimal T cell–dependent vaccine responses. A broader impact on the GALT–splenic axis is also plausible, potentially supporting T cell–independent responses to polysaccharide antigens. Together, these findings underscore the central role of complement in orchestrating intestinal and systemic immunity and highlight the transformative impact of complement inhibition in restoring B cell homeostasis in CHAPLE disease.

## Materials and methods

### Patients

In this study, we enrolled eight patients from seven unrelated kindreds, all residing in Algeria. Seven patients were of Algerian ancestry, and one was of Syrian ancestry. Patients were initially identified based on a diagnosis of persistent PLE and were subsequently included upon confirmation of a defect in CD55 expression via flow cytometry (FCM), together with the identification of biallelic LOF variants in the *CD55* gene. Data were systematically collected for each patient using a standardized data form and uploaded to a computerized database. Information included demographics, family history, clinical information, and laboratory, imaging, and endoscopic findings. All participants provided informed consent, and the study was approved by the local ethics committee, in accordance with the Declaration of Helsinki.

### Serum proteins measurement and CD55 expression analysis

Serum albumin, complement C3 and C4, and immunoglobulin (IgG, IgM, and IgA) levels were measured by nephelometry (BN ProSpec System, Siemens). CD55 expression was analyzed by FCM as previously described (34). Briefly, whole blood from patients, healthy controls, and family members was stained with CD15-APC, CD45-APC-H7, and CD55-PE, or the corresponding isotype control for 20 min at room temperature in the dark. Following erythrocyte lysis and washing, samples were acquired on an eight-color FACSLyric cytometer (BD Biosciences). CD55 expression on neutrophils was quantified both as the percentage of positive cells and as MFI, and results were compared with those from healthy controls.

### Genetic testing and structural modeling

WES was performed in all patients as part of the diagnostic workup for CHAPLE disease using an Illumina sequencing platform. Sequence alignment and variant calling were carried out using a validated bioinformatics pipeline. Variant filtering focused on rare variants with low allele frequency in population databases, including gnomAD, and on predicted deleterious variants consistent with a LOF effect in CD55. Variant pathogenicity was further supported by in silico prediction tools, including Combined Annotation Dependent Depletion scores. Candidate variants were interpreted according to the American College of Medical Genetics and Genomics guidelines. To further assess the potential structural impact of the identified *CD55* variant, in silico structural modeling was performed. Models were visualized and analyzed using UCSF ChimeraX v1.10.1, based on the available crystal structure (UniProt ID: 1OJV) and an AlphaFold-predicted model (UniProt ID: P08174-4). Local structural features and conformational flexibility were further evaluated using ColabFold, B-factor calculations in ChimeraX, and the DynaMut web server.

### Lymphocyte phenotyping

For each patient, basic immunophenotyping of circulating lymphocyte subsets, referred to as T-B-NK enumeration, was performed. In addition, advanced FCM analysis was performed to enable comprehensive characterization of circulating T and B cell subsets, as previously described (34). Briefly, a six-color panel was used for T cells, incorporating CD3, CD4, CD8, CD45RA, CD45RO, and CCR7 (CD197) markers, enabling the identification of naïve (CD45RA^+^CCR7^+^) and memory (CD45RO^+^) subsets among CD4^+^ or CD8^+^ T cells. A multicolor panel, including CD19, CD27, surface IgD, CD24, CD38, and CD21 markers, was used to analyze different B cell subsets, including naïve (CD27^–^IgD^+^), unswitched memory (CD27^+^IgD^+^), switched memory (CD27^+^IgD^–^), transitional (CD24^++^CD38^++^), plasmablasts (CD24^–^CD38^++^), and CD21-low (CD21^lo^) B cells (CD21^low^CD38^low^).

### Postvaccinal assessment of pneumococcal serotype–specific IgG responses

Prior to eculizumab therapy, patients P5, P6, and P8 received meningococcal and pneumococcal vaccines. Post-pneumococcal serotype–specific IgG responses were assessed following sequential vaccination with PCV13 (Prevenar 13) and PPSV23 (Pneumovax 23), with PCV13 administered first and PPSV23 2 mo later. Eculizumab was initiated 15 days after PPSV23 in patients P6 and P8, whereas in patient P5 it was administered 3 mo after PPSV23. Blood samples were collected at defined time points. Patient P5 had samples at T0 (pre-vaccination), T1 (60 days after sequential PCV13 and PPSV23 vaccination, prior to eculizumab), and T2 (60 days after eculizumab initiation). Patient P6 had a single post-vaccination sample 2 mo after eculizumab initiation. Patient P8 had samples at T0 (pre-vaccination) and T2 (60 days after eculizumab initiation). Serotype-specific IgG concentrations were measured using a quantitative multiplex chemiluminescent immunoassay, allowing simultaneous detection of IgG antibodies against 11 pneumococcal serotypes included in PCV13 (1, 3, 4, 5, 6B, 7F, 9V, 14, 18C, 19F, and 23F) and additional serotypes present exclusively in PPSV23 (8, 9N). Individual serotype concentrations ≥1.3 µg/ml were considered protective.

### Online supplemental material

Supplementary materials include Fig. S1, showing the markers and gating strategy used for B and T cell immunophenotyping, with representative FCM analyses from patient P6 and an age-matched healthy control; Table S1, summarizing the laboratory and immunophenotypic findings of the eight patients with CHAPLE disease; and Table S2, detailing the dynamics of pneumococcal serotype–specific IgG concentrations following sequential PCV13 and PPSV23 vaccination and eculizumab therapy in patients P5, P6, and P8.

## Supplementary Material

Table S1 shows laboratory and immunophenotypic findings of eight patients with CHAPLE disease.

Table S2 shows dynamics of pneumococcal serotype–specific IgG concentrations following sequential PCV13 and PPSV23 vaccination and eculizumab therapy.

## Data Availability

Data are available from the corresponding authors upon reasonable request.
