# Supplementary material for: C5 inhibition restores B cell homeostasis and humoral immunity in CHAPLE disease patients
Source: J Hum Immun. 2026 May 26;2(4):e20260042. doi: 10.70962/jhi.20260042 (PMC13205138; doi:10.70962/jhi.20260042)
Supplement: Table S1 — shows laboratory and immunophenotypic findings of eight patients with CHAPLE disease. [file jhi_20260042_tables1.docx]

**Table S1:** Laboratory and immunophenotypic findings of 8 patients with CHAPLE disease.

| Parameter | **P1** | **P2** | **P3** | **P4** | **P5** | **P6** | **P7** | **P8** |
| --- | --- | --- | --- | --- | --- | --- | --- | --- |
| **Age** at evaluation, years | 5 | 7 | 6 | 2 | 15 | 5 | 8 | 11 |
| **Albumin**, g/L  [NV: 32 – 45] | **15** | **16** | **21** | **23** | **19** | **12** | **27** | **23** |
| *Complement proteins* | | | | | | | | |
| **C3**, mg/dL  [NV: 89 – 187] | 92 | 118 | 91 | 146 | **82** | 89 | 93 | **82** |
| **C4**, mg/dL  [NV: 16 – 38] | 26 | **11** | 39 | 29 | 20 | 22 | 28 | 21 |
| *Immunoglobulins, mg/dL [NV]* | | | | | | | | |
| **IgG** | **151**  [477 – 1,551] | **170**  [565 – 1,520] | 553  [477 – 1,551] | 532  [310 – 1,200] | **178**  [610 – 1,480] | **122**  [477 – 1,551] | 692  [565 – 1,520] | **201**  [565 – 1,520] |
| **IgM** | **24**  [40 – 200] | **10**  [50 – 180] | 47  [40 – 200] | 62  [45 – 150] | **30**  [50 – 190] | **32**  [40 – 200] | **47**  [50 – 180] | **26**  [50 – 180] |
| **IgA** | 48  [40 – 200] | **34**  [50 – 240] | 184  [40 – 200] | **161**  [35 – 80] | **74**  [80 – 280] | 51  [40 – 200] | 171  [50 – 240] | **41**  [50 – 240] |
| *Lymphocyte phenotyping* | | | | | | | | |
| **Lymphocytes**, cells/μL [NV] | **1,430**  [2,300 – 5,400] | **4,206**  [1,900 – 3,700] | **1,732**  [2,300 – 5,400] | 7,087  [3,600 – 8,900] | **5,166**  [1,400 – 3,300] | **6,237**  [2,300 – 5,400] | **1,239**  [1,900 – 3,700] | **3,758**  [1,900 – 3,700] |
| *T-B-NK enumeration, cells/μL [NV]* | | | | | | | | |
| **CD3^+^ T cells** | **772**  [1,400 – 3,700] | **3,533**  [1,200 – 2,600] | **866**  [1,400 – 3,700] | 3,898  [2,100 – 6,200] | **4,340**  [1,000 – 2,200] | **4,304**  [1,400 – 3,700] | **756**  [1,200 – 2,600] | **2,856**  [1,200 – 2,600] |
| **CD4^+^ T cells** | **372**  [700 – 2,200] | **1,767**  [650 – 1,500] | **589**  [700 – 2,200] | 2,551  [1,300 – 3,400] | **2,170**  [530 – 1,300] | **2,245**  [700 – 2,200] | **235**  [650 – 1,500] | **1,654**  [650 – 1,500] |
| **CD8^+^ T cells** | **365**  [490 – 1,300] | **1,598**  [370 – 1,100] | **225**  [490 – 1,300] | 1,063  [620 – 2,000] | **2,015**  [330 – 920] | **1,559**  [490 – 1,300] | 434  [370 – 1,100] | 940  [370 – 1,100] |
| **CD4/CD8 ratio** | 1.02 | 1.11 | 2.62 | 2.40 | 1.08 | 1.44 | **0.54** | 1.76 |
| **CD19^+^** **B cells** | **243**  [390 – 1,400] | 379  [270 – 860] | 398  [390 – 1,400] | 1,347  [720 – 2,600] | 207  [110 – 570] | 686  [390 – 1,400] | **87**  [270 – 860] | 752  [270 – 860] |
| **NK cells** | 415  [130 – 720] | 252  [100 – 480] | 433  [130 – 720] | **1,843**  [180 – 920] | **620**  [70 – 480] | **1,247**  [130 – 720] | 335  [100 – 480] | 113  [100 – 480] |
| *Extended CD4^+^ T-cell phenotyping, % of CD4 (cells/μL) / [NV]* | | | | | | | | |
| CD4^+^CD45RA^+^ | **35 (130)**  [430 – 1,500] | **38** (671)  [320 – 1,000] | **41 (241)**  [430 – 1,500] | 71 (1,811)  [1,000 – 2,900] | 48 **(1,042)**  [230 – 770] | 66 (1,482)  [430 – 1,500] | **33 (78)**  [320 – 1,000] | 51 (844)  [320 – 1,000] |
| **naïve CD4^+^ T cells** (CD45RA^+^CCR7^+^) | **32 (119)**  [420 – 1,500] | **31** (548)  [310 – 1,000] | **39 (230)**  [420 – 1,500] | 69 (1,760)  [950 – 2,800] | 46 **(998)**  [210 – 750] | 65 (1,459)  [420 – 1,500] | **32 (75)**  [310 – 1,000] | 50 (827)  [310 – 1,000] |
| **memory CD4^+^ T cells**  (CD45RO^+^) | **65** (242)  [220 – 660] | **62 (1,096)**  [230 – 630] | **59** (348)  [220 – 660] | 29 (740)  [210 – 850] | 52 **(1,128)**  [240 – 700] | 34 **(763)**  [220 – 660] | **67 (157)**  [230 – 630] | 49 **(810)**  [230 – 630] |
| *Extended CD8^+^ T-cell phenotyping, % of CD8 (cells/μL / [NV]* | | | | | | | | |
| CD8^+^CD45RA^+^ | **65 (237)**  [380 – 1,100] | 63 **(1,007)**  [310 – 900] | 82 **(185)**  [380 – 1,100] | 89 (946)  [490 – 1,700] | **58** **(1,169)**  [240 – 710] | 66 (1,029)  [380 – 1,100] | **44 (191)**  [310 – 900] | **49** (461)  [310 – 900] |
| **naïve CD8^+^ T cells**  (CD45RA^+^CCR7^+^) | **20 (73)**  [260 – 850] | **23** (368)  [200 – 650] | 56 **(126)**  [260 – 850] | 75 (797)  [400 – 1,400] | **37** **(746)**  [170 – 560] | 61 **(951)**  [260 – 850] | **16 (69)**  [200 – 650] | **32** (301)  [200 – 650] |
| **memory CD8^+^ T cells**  (CD45RO^+^) | 35 (128)  [90 – 440] | 37 **(591)**  [70 – 390] | 18 **(41)**  [90 – 440] | 11 (117)  [60 – 570] | **42** **(846)**  [60 – 310] | 34 **(530)**  [90 – 440] | **56** (243)  [70 – 390] | **51 (479)**  [70 – 390] |
| *Extended CD19^+^ B-cell phenotyping, % of CD19 (cells/μL) / [NV]* | | | | | | | | |
| **naïve B cells**  (CD27^–^IgD^+^) | **47.0 (114)**  [334 – 611] | **43.9** (166)  [133 – 389] | 72.8 (290)  [133 – 389] | **80** (1,078)  [571 – 1,323] | **23.2 (48)**  [171 – 293] | **41.7 (286)**  [334 – 611] | **41.8 (36)**  [133 – 389] | **66.1 (497)**  [171 – 293] |
| **unswitched memory B cells**  (CD27^+^IgD^+^) | **16.2** (39)  [25 – 60] | **21.0 (80)**  [22 – 43] | 4.1 **(16)**  [22 – 43] | **4.0** (54)  [38 – 90] | **25.5 (53)**  [12 – 32] | **19.9 (137)**  [25 – 60] | **18.7 (16)**  [22 – 43] | **10.6 (80)**  [12 – 32] |
| **switched memory B cells**  (CD27^+^IgD^–^) | **26.8 (65)**  [16 – 44] | **31.0 (117)**  [16 – 31] | **11.0 (44)**  [16 – 31] | **6.0 (81)**  [13 – 42] | **41.5 (86)**  [10 – 29] | **22.5 (154)**  [16 – 44] | **29.0** (25)  [16 – 31] | **14.7 (111)**  [10 – 29] |
| **Transitional B cells**  (CD24^++^CD38^++^) | **0.8 (2)**  [23 – 71] | **1.0 (4)**  [13 – 36] | **0.5 (2)**  [13 – 36] | **1.0 (13)**  [56 – 101] | **0.4 (1)**  [10 – 24] | **1.0 (7)**  [23 – 71] | **0.8 (1)**  [13 – 36] | **0.9 (7)**  [10 – 24] |
| **CD21^lo^ B cells**  (CD21^low^CD38^low^) | **7.8** (19)  [8 – 23] | 2.8 **(11)**  [3 – 4] | **7.0 (28)**  [3 – 4] | **8.0 (108)**  [15 – 36] | **10.7 (22)**  [2 – 12] | **7.6 (52)**  [8 – 23] | **4.8 (4)**  [3 – 4] | **5.2 (39)**  [2 – 12] |
| **Plasmablasts**  (CD24^–^CD38^++^) | **3.6** (9)  [5 – 15] | 0.7 (3)  [2 – 12] | **7.0 (28)**  [2 – 12] | **3.0 (40)**  [4 – 19] | **6.2 (13)**  [1 – 4] | **10.9 (75)**  [5 – 15] | 2.6 (2)  [2 – 12] | **3.7 (28)**  [1 – 4] |

Bolded values indicate results outside the normal reference range. Normal values (NV, shown in brackets) are expressed as absolute cell counts (cells/µL) **(12, 34)**.
