# Supplementary material for: C5 inhibition restores B cell homeostasis and humoral immunity in CHAPLE disease patients
Source: J Hum Immun. 2026 May 26;2(4):e20260042. doi: 10.70962/jhi.20260042 (PMC13205138; doi:10.70962/jhi.20260042)
Supplement: Table S2 — shows dynamics of pneumococcal serotype–specific IgG concentrations following sequential PCV13 and PPSV23 vaccination and eculizumab therapy. [file jhi_20260042_tables2.docx]

**Table S2:** Dynamics of pneumococcal serotype–specific IgG concentrations following sequential PCV13 and PPSV23 vaccination and eculizumab therapy.

| **Patient** | **Timepoint** | **PCV13 serotypes**  **(µg/mL)** | | | | | | | | | | | **PPSV23-only serotypes**  **(µg/mL)** | |
| --- | --- | --- | --- | --- | --- | --- | --- | --- | --- | --- | --- | --- | --- | --- |
|  |  | **Pneumo**  **1** | **Pneumo**  **3** | **Pneumo**  **4** | **Pneumo**  **5** | **Pneumo**  **6B** | **Pneumo**  **7F** | **Pneumo**  **9V** | **Pneumo**  **14** | **Pneumo**  **18C** | **Pneumo**  **19F** | **Pneumo**  **23F** | **Pneumo**  **8** | **Pneumo 9N** |
| **P5** | T0 | 0.14 | 0.01 | 0.03 | 0.04 | 0.01 | 0.19 | 0.01 | 0.05 | 0.01 | 0.01 | 0.01 | 0.08 | 0.01 |
|  | T1 Post-vaccines | 0.22 | 0.01 | 0.02 | 0.04 | 0.01 | 0.34 | 0.01 | 0.12 | 0.01 | 0.28 | 0.01 | 0.16 | 0.01 |
|  | T2 Post-eculizumab | 1.65 | 0.26 | 0.20 | 0.11 | 0.21 | 2.66 | 0.14 | 1.80 | 0.19 | 3.02 | 0.08 | 1.81 | 0.10 |
| **P6** | Post-vaccines + eculizumab | 7.46 | 6.68 | 11.94 | 9.33 | 7.85 | 8.65 | 9.73 | 8.92 | 4.92 | 112.96 | 9.46 | 0.86 | 5.18 |
| **P8** | T0 | 0.01 | 0.01 | 0.01 | 0.05 | 0.01 | 0.01 | 0.01 | 0.03 | 0.01 | 0.01 | 0.03 | 0.03 | 0.01 |
|  | T1 Post-vaccines + eculizumab | 1.59 | 4.11 | 8.18 | 3.51 | 3.83 | 3.36 | 4.92 | 14.79 | 6.62 | 10.76 | 0.98 | 1.60 | 2.38 |

***Abbreviations:*** *PCV13, 13-valent pneumococcal conjugate vaccine; PPSV23, 23-valent pneumococcal polysaccharide vaccine.*
